# Barriers to Implementing Evidence-Based Practice among Primary Healthcare Nurses in Saudi Arabia: A Cross-Sectional Study

**DOI:** 10.3390/nursrep12020031

**Published:** 2022-04-28

**Authors:** Jamaan M. Alqahtani, Rene P. Carsula, Homood A. Alharbi, Seham M. Alyousef, Omar G. Baker, Regie B. Tumala

**Affiliations:** 1 Nursing Affairs, Ministry of Health, Riyadh 11176, Saudi Arabia; jamalqahtani@moh.gov.sa; 2 Community and Mental Health Nursing Department, College of Nursing, King Saud University, Riyadh 12372, Saudi Arabia; rcarsula@ksu.edu.sa (R.P.C.); smansour@ksu.edu.sa (S.M.A.); obaker@ksu.edu.sa (O.G.B.); 3 Medical-Surgical Nursing Department, College of Nursing, King Saud University, Riyadh 12372, Saudi Arabia; homalharbi@ksu.edu.sa

**Keywords:** barrier, evidence-based practice, nurse, primary healthcare center, Saudi Arabia

## Abstract

Several studies have been conducted to investigate the barriers to implementing evidence-based practice (EBP) worldwide. In Saudi Arabia, a few studies were conducted in hospital and mental health settings, and no study has explored the topic in primary healthcare settings. This study aimed to examine the barriers perceived by primary healthcare nurses in implementing EBP. This study employed a correlational and cross-sectional design. A total of 284 nurses were surveyed using the BARRIERS scale. Regression analysis was performed to identify the effects of predictor variables on the four subscales. The overall raw score on the BARRIERS scale was 86.21 (standard deviation = 16.15). The highest mean score was reported in the organizational subscale, followed by the innovation and communication subscales, whereas the lowest mean score was reported in the adopter subscale. The findings showed that the three top-ranked barriers were: (1) results of the studies are not generalizable to nurses’ setting, (2) facilities are inadequate, and (3) physicians do not cooperate with the implementation. Findings showed that nurses encounter organizational-related barriers to a moderate extent and EBP implementation varies depending on gender, level of education, and job position. There is a need to create and implement educational interventions and programs to overcome the barriers to effective EBP implementation among primary healthcare nurses.

## 1. Introduction

Evidence-based practice (EBP) is considered a key factor in improving the quality of healthcare and patient outcomes [1,2,3]. EBP is a combination of three core components: (1) recent best available evidence, (2) expertise and analysis of the clinician, and (3) values, preferences, views, and expectations of the patient [4,5]. In other words, EBP refers to the integration of clinical expertise with patients’ preferences and values and the best available evidence by translating informational needs into answerable questions and then tracking the best information to answer the questions [5]. In addition, the practice of EBP is influenced by the level of the available evidence, the clinical experience of healthcare providers, and the patients’ desires and expectations [4,5,6]. 

In nursing practice, EBP enhances nurses’ decision-making ability and improves their ability to formulate individualized care plans that lead to efficient patient care [7]. The literature indicates that nurses value EBP; however, its implementation is inconsistent because it is often obstructed by many factors, such as paucity of facilities, time, resources, support, knowledge and skills, autonomy, and funding, as well as access limitations, which have been reported in several studies conducted globally [1,8,9,10,11,12,13,14,15,16,17,18,19]. This evidence shows that for several years, there has been a wide gap between research and practice brought about by barriers to EBP implementation.

For decades, the unceasing challenges for all organizations have been the regular evaluation of research and the utilization, prioritization, and dissemination of research findings for application in clinical practice [20]. In the UK, McKenna et al. [15] conducted a study on 356 primary care nurses and identified the main barriers to EBP, namely organizational issues regarding cost, changes in work, time limitations, patient compliance, and lack of motivation to use EBP. Youssef et al. [11] indicated that both Egyptian and Jordanian nurse educators hold positive attitudes toward EBP adoption; however, they encountered several barriers to the EBP implementation processes. In Iran, Khammarnia et al. [13] conducted a cross-sectional study in Zahedan with 280 nurses and found that the majority of the respondents agreed that the barriers to EBP implementation are related to individual and organizational factors. Al-Maskari and Patterson [14] also conducted a descriptive cross-sectional study in three governmental hospitals involving 282 registered nurses in Oman. They indicated that compared with staff nurses, nurse leaders had higher scores in changing practice and finding and reviewing evidence of the barrier subsections, with lower scores for the facilitators to changing the practice section.

In Saudi Arabia, Hamaideh [19] noted that the barriers to using EBP include lack of time to find research outputs, insufficient availability of resources, and difficulty in understanding research findings. In addition, Alqahtani et al. [1] conducted the most recent study involving 227 staff nurses in four hospitals in Riyadh. The study revealed that 36.6% of the respondents reported that workload, lack of time, lack of skills and knowledge, lack of organizational communication and dissemination of information on EBP, fear of mistakes and error, colleagues’ resistance to change, and lack of financial support prevented them from implementing EBP in their practice. Due to this and the lack of empirical research on barriers to EBP implementation in primary healthcare settings, and particularly, to date, no study has investigated the topic in Saudi Arabia; this study could help fill the gap between research and practice in the literature. Hence, the present study examined the perceived barriers of nurses and their predictors to EBP adoption in primary healthcare centers in Riyadh, Saudi Arabia.

## 2. Materials and Methods

### 2.1. Study Design, Setting, and Sampling

This descriptive, quantitative study used a cross-sectional, correlational design. The study adhered to the Strengthening the Reporting of Observational studies in Epidemiology (STROBE) guidelines for cross-sectional studies [21]. 

The respondents were recruited through convenience sampling to achieve wide participation among staff nurses, assistant nurse managers, and nurse managers working in 50 randomly selected primary healthcare centers in the city of Riyadh, Saudi Arabia. The criteria for inclusion in this study are as follows: (1) a licensed nurse in Saudi Arabia, (2) currently working in one of the selected primary healthcare centers in Riyadh City, (3) with at least one-year experience in primary nursing care, and (4) consented to participate. Nurses working in academic and hospital settings and Saudi nurses continuing their education overseas were excluded from participating in the study. 

The sample size was calculated through a G*Power 3.1.9 application [22]. The alpha was assigned with a value of 0.05, the confidence interval was 0.95, the effect size was 0.3 (medium), and the study sample of 284 primary healthcare nurses was adequate. 

### 2.2. Instrumentation

The questionnaire used in this study has two parts. The first part investigated the demographic characteristics of the respondents, such as age, gender, level of educational attainment, length of service in years, job position, and attendance in any training or courses related to EBP. The second part incorporated the BARRIERS scale–the Barriers to Research Utilization Scale, which is composed of 29 items. The scale was originally developed at the University of North Carolina in the US [23]. Permission to use the scale was obtained from the copyright holder. The respondents in this study were asked to rate the items on the BARRIERS scale using five-point Likert-type responses that indicate: (1) to no extent; (2) to a little extent; (3) to a moderate extent; (4) to a great extent; and (5) no opinion. The scale scores ranged from 29 to 145. The four subscales used in this study were based on the factor-analytic procedures of Funk et al. [23], namely (1) adopter, which pertains to nurse’s values, skills, and awareness concerning the research; (2) organization, which pertains to the barriers and limitations related to the setting; (3) innovation, which pertains to the quality of the research; and (4) communication, which pertains to the presentation and accessibility of the research. The scale demonstrated high face and content validity, with Cronbach’s alpha coefficients ranging from 0.65 to 0.80 for the four-factor groups [23].

### 2.3. Ethical Consideration and Data Collection

Before conducting the study, two ethical approvals were obtained. In addition, administrative permission was obtained from the president’s office of the primary healthcare sectors. Throughout the study, the researchers adhered to the guidelines and ethical standards while working with human respondents required by the Institutional Review Boards and the Declaration of Helsinki and its revisions.

Consent from the respondents was obtained, and the confidentiality of all collected data was ensured. The respondents were recruited through verbal invitations and posters with the facilitation of the nurse managers. The self-administered questionnaires were distributed to the nurses in their working areas after a briefing and orientation on the purpose of the study. After completing the questionnaires, the respondents were asked to place the questionnaires in the designated envelopes and boxes. Data collection was done between June 2019 and July 2019.

### 2.4. Statistical Analysis

The data were analyzed using SPSS for IBM version 23 (Armonk, NY, USA). Descriptive statistics (i.e., frequency, mean, median, and standard deviation) were used to describe the respondents’ responses to barriers to implementing EBP. A *t*-test was performed to determine the differences in the perceptions of nurses regarding barriers to applying EBP according to demographic characteristics. Multiple regression analysis was performed to identify the demographic variables associated with the nurses’ barriers to EBP adoption. Findings were considered significant if *p* < 0.05. 

## 3. Results

### 3.1. Characteristics of the Respondents

The study was conducted in 50 primary healthcare centers in Riyadh City, Saudi Arabia. There were 350 questionnaires distributed, and 291 were returned, of which seven were excluded due to incomplete data; therefore, a total of 284 surveys were included, indicating a response rate of 81.1%. The demographic characteristics of the respondents revealed that most of the nurses, 73.6%, were female, with 66.5% holding a diploma in nursing, 67.3% being staff nurses, and 57.7% with no previous EBP training. The respondents’ age ranged from 23 to 55 years (mean = 34.69 years old; SD = 6.38), and their years of experience ranged from 1 to 36 years (mean = 12 years; SD = 6.91).

### 3.2. Nurses’ Raw Scores of Barriers to Implementing EBP

The raw scores of the perceptions of primary healthcare nurses regarding the barriers to EBP implementation showed that the lowest score was 35, and the highest was 124. The overall mean (86.21; SD = 16.15) and median (87) scores are close to each other, indicating that the nurses experienced a moderate extent of the barriers to implementing EBP in the primary healthcare setting. Similarly, the results exhibited that the majority of the respondents reported experiencing a moderate extent of barriers to implementing EBP (52.3%), 37.7% encountered a great extent of the EBP barriers, only 5.7% experienced barriers to a small extent, and 3.3% had no opinion about EBP barriers.

### 3.3. Nurses’ Perceived Barriers to Implementing EBP

Table 1 shows that the organization subscale has the highest overall mean score (mean = 3.14; SD = 0.66), followed by the innovation subscale (overall mean = 2.98; SD = 0.72) and the communication subscale (overall mean = 2.96; SD = 0.66). In contrast, the adopter subscale has the lowest overall mean score (mean = 2.87; SD = 0.65). Moreover, in the organization subscale, the item “the nurse feels results are not generalizable to own setting” has the highest mean score (mean = 3.39; SD = 0.05), whereas the item “other staff are not supportive of implementation” has the lowest mean score (mean = 2.92; SD = 1.15). In the innovation subscale, the item “research reports/articles are not published fast enough” has the highest mean score (mean = 3.12; SD = 1.05), whereas the item “the nurse is uncertain whether to believe the results of the research” has the lowest mean score (mean = 2.72; SD = 1.18). In the communication subscale, the item “the relevant literature is not compiled in one place” has the highest mean score (mean = 3.23; SD = 1.06), whereas the item “the amount of research information is overwhelming” has the lowest mean score (mean = 2.84; SD = 1.09). Meanwhile, in the adopter subscale, the item “the nurse feels the benefits of changing practice will be minimal” has the highest mean score (mean = 3.10; SD = 1.05), whereas the item “the nurse is unwilling to change/try new ideas” has the lowest mean score (mean = 2.31; SD = 1.15).

In exploring the top-ranked and lowest-perceived EBP barriers, the results showed the 10 top-ranked and 3 lowest-rated barriers identified by the nurses in implementing EBP (Table 1). Out of the 10 top-ranked items rated by the respondents, half (50%) of the items were from the organization subscale, 2 items were from the adopter subscale, 2 items were from the innovation subscale, and 1 item was from the communication subscale. The 5 items from the organization subscale include the following: “the nurse feels results are not generalizable to own setting” (mean = 3.39; SD = 1.05), “the facilities are inadequate for implementation” (mean = 3.25; SD = 0.94), “physicians will not cooperate with implementation” (mean = 3.24; SD = 0.99), “the nurse does not feel she/he has enough authority to change patient care procedures” (mean = 3.17; SD = 1.01), and “the nurse does not have time to read research” (mean = 3.09; SD = 1.04). Meanwhile, the 3 lowest-rated barriers indicated that most of the nurses were willing to change and try new ideas (mean = 2.31; SD = 1.15), see the value of research for practice (mean = 2.69; SD = 1.29), and certainly believe in the results of the research (mean = 2.72; SD = 1.18).

### 3.4. Differences of BARRIERS Scale Raw Scores and Nurses’ Demographic Characteristics

The differences in the raw scores of the barriers and the demographic variables of the respondents, including age, gender, level of education, job position, years of experience, and EBP training, showed no significant results as the *p*-values were higher than 0.05 (Table 2).

### 3.5. Results of Regression Analysis on the BARRIERS Subscales

The overall mean scores of the nurses in the barrier subscales were entered into a regression analysis model with the demographic characteristics as the predictor variables. Table 3 shows the regression model with negatively significant results on nurses’ gender (β = −0.21, *p* = 0.02, 95% CI [−0.035, −0.405]) and level of education (β = −0.297, *p* = 0.003, 95% CI [−0.097, −0.472]) in the adopter subscale, and on nurses’ job position in the communication subscale (β = −0.235, *p* = 0.01, 95% CI [−0.061, −0.486]).

## 4. Discussion

This study examined the existence of barriers to EBP implementation in primary healthcare nursing in Riyadh City, Saudi Arabia. The study revealed critical findings indicating that most primary healthcare nurses experienced a moderate extent of the barriers to implementing EBP with an overall raw score of 86.21. Specifically, the results exhibited that the majority of the respondents reported experiencing a moderate extent of barriers to implementing EBP (52.3%), 37.7% encountered a great extent of barriers, and only 5.7% experienced a little extent of EBP barriers. Notably, 3.3% of the respondents had no opinion about EBP barriers. The latter finding is similar to that reported in a recent study conducted in Saudi Arabia, where 8.8% of the 227 hospital nurses reported that they are not facing any barrier in implementing EBP [1]. Although the overall raw score in this study is higher than that reported in the study by Bayik et al. [18] in Turkey (overall raw score = 75.13), both studies reported that nurses encountered a moderate extent of the barriers in EBP implementation. In addition, the highest mean score (3.15) was reported on the item “the facilities are inadequate for implementation” in the previous study [18], whereas in this study, the same barrier item was ranked second with a mean score of 3.25 (higher than that of the previous study). On comparing the results of this study with those of the previous studies [15,16], it was observed that the primary healthcare nurses in Saudi Arabia likely consider the inadequacy of facilities as a substantial barrier compared with the nurses in the UK and Maldives. Zhou et al. [9] conducted another study among Chinese nurses and found that the top-ranked barrier reported was the lack of time on the job to read the research, which belongs to the organization subscale. Similar findings were also indicated in previous studies conducted in Iran [10] and Oman [14].

Labrague et al. [24] stated that clinical settings and their organizations play critical and important roles in the successful implementation and integration of EBP. However, the results in this study revealed that 5 out of the 10 top-ranked barriers belong to the organization subscale. These organizational barriers include that primary healthcare nurses do not have time to read research, they feel that research results are not generalizable to their own organization, and they do not have sufficient authority to change patient care procedures based on evidence-based research. The respondents also reported that primary healthcare physicians are not cooperative, and the facilities in the primary healthcare centers are not adequate for EBP implementation. Similarly, among the four subscales, the highest mean score (3.14) was reported in the organization subscale. This result is consistent with a systematic review of 63 studies where the setting/organization is identified as the main barrier to EBP adoption [25]. These findings suggest that the major source of barriers as perceived by nurses in this study is their organizations in the primary healthcare center clusters in Riyadh. 

The results of this study are contradictory to the results reported in studies from England, where an organization is committed to adopting EBP at the topmost level when senior practice nurses have the autonomy to develop their role in recognizing innovative opportunities to promote EBP at the frontline [10]. Comparably, the results of a Canadian study that used the mixed method design indicated that nurses identified contextual barriers related to time availability and material resources, which contributed to their negative perceptions of EBP interventions for managing patient-oriented outcomes [8]. However, as reported in this study, the organizational-related barriers to EBP adoption, such as having lack of time and resources as well as not having the autonomy to change practice, and other barriers, including infrastructure, administrative support, and facilities, have been consistently reported in previous studies and reviews [26,27,28]. In a study in the USA, the barriers to implementing EBP remain prevalent among nurses, which include resistance from colleagues, nurse leaders, and managers [17]. Similarly, in Saudi Arabia, the fear of committing mistakes, the resistance from colleagues, and the lack of time, resources, EBP skills and knowledge, financial support, and dissemination of EBP in the organization prevented nurses from implementing EBP into their practice [1]. In addition, the barriers to EBP implementation, as reported by Saudi mental health nurses, were lack of time to find research outputs, insufficient availability of resources, and difficulty in understanding research findings [19]. 

The value of nurses in adopting EBP and their willingness to change and try new ideas have been reported in this study as the least perceived EBP barriers. These lowest-ranked barriers belong to the adopter subscale. Nurses also claimed that they certainly believed in the results of the research. These findings are consistent with and supported by the results of a recent multi-country study among nursing students in India, Nigeria, Oman, and Saudi Arabia [24]. In particular, the finding of another study in Saudi Arabia supports this finding of the need to incorporate EBP concepts into the nursing curricula and clinical practice [29].

The results of this study showed no significant differences between the raw scores of the barriers and the demographic data (i.e., age, gender, level of education, job position, years of experience, and EBP training) of the respondents. In contrast, in another study, differences were found in the responses of US nurses between those with and without a master’s degree [17]. Meanwhile, the relationship between the barrier subscales and nurses’ demographic data revealed significant predictions where the nurses’ gender negatively predicted the extent of EBP barriers encountered by nurses in the adopter subscale. This finding indicates that male nurses reported a higher extent of EBP barriers in the adopter subscale compared with their female counterparts. The results might be related to the fact that three-quarters of the sample were females, as expected in the primary healthcare centers in Saudi Arabia due to gender segregation [30], where female sections have more clinics than their male counterparts (e.g., maternity and child health departments).

In addition, the educational level of the respondents negatively predicted the extent of EBP barriers encountered by nurses in the adopter subscale. This finding indicates that nurses with a Bachelor’s degree in Nursing exhibited a higher extent of EBP barriers in the adopter subscale compared with those who had a Diploma in Nursing. The result is noteworthy given that nurses with a Bachelor’s degree in Nursing could have exhibited a lower extent of EBP barriers compared with those who had a Diploma in Nursing because they have studied EBP concepts in their research courses. Moreover, the lesser number of nurses with a Bachelor’s degree in Nursing is noticeable and expected in most of the primary healthcare centers because the general directorate of health in all regions in Saudi Arabia considers and assigns them critically and suitably in secondary and tertiary healthcare institutions. This case may impact the application of EBP in primary healthcare settings at regional and national levels. Moreover, the job position of the respondents negatively predicted the extent of EBP barriers encountered by nurses in the communication subscale. This finding shows that nurse managers encountered a higher extent of EBP barriers in the communication subscale compared with their staff nurses. The findings of this study are comparable to those of another Omani study, where nurse leaders showed higher item mean scores for the barriers to EBP implementation than their staff nurses [14].

Kajermo et al. [25] noted that the findings of their systematic review involving 63 studies that investigated the correlations between demographic characteristics (e.g., age, education, and professional experience) and perceptions of EBP barriers were inconclusive. Furthermore, the authors indicated that obtaining a distinctive image of the correlations is difficult because demographic data are often presented in diverse ways and are associated with the subscales or the individual items [25]. In the current study, the major barrier perceived by the respondents is linked to the organization subscale. However, no significant associations with nurses’ demographic characteristics were indicated. In contrast, an association between the organization subscale and knowledge and skills with EBP was reported by Brown et al. [26]. In contrast, clinical and research experience, job satisfaction, and working pressure were identified as associated factors for Chinese nurses’ barriers to their EBP adoption [9]. Finally, another Iranian study revealed that age, level of education, job experience, and employment status were associated with organizational barriers. The level of education was associated with adopter barriers, and the barriers occurred at the adopter and organizational subscales [13].

## 5. Conclusions and Implications for Nursing Practice

The results of this current study showed that nurses had encountered organizational-related barriers (e.g., nurses’ lack of time to read research, ungeralizability of research findings to nurses’ organization, nurses’ insufficient authority to change patient care procedures, uncooperative primary healthcare physicians, and inadequate facilities) to EBP implementation to a moderate extent. This indicates that these barriers have prevented them from adopting EBP in their professional practice in primary healthcare settings. However, the nurses mentioned that they see the value of research, believe in the results of research for practice, and are willing to change or try new ideas for implementing EBP. In addition, the study provides evidence that barriers to EBP implementation vary among primary healthcare nurses depending on gender, level of education, and job position.

Primary healthcare nurses must find and allocate time to read and use research on and maintain EBP implementation as they practice in primary healthcare settings. They must find possible ways and means to adopt evidence-based research findings that may guide their day-to-day practice in primary healthcare centers. Ensuring this will enable nurses to utilize research to adopt EBP to deliver high-quality patient care. In addition, nursing administrators at the national, regional (i.e., Arab speaking countries), and international levels, must support and involve nurses under their leadership to plan and implement educational interventions and activities for overcoming the organizational barriers to EBP adoption at all time points. Likewise, medical administrators and authorities of the primary healthcare center clusters from the Ministry of Health must be consulted and involved in the process of planning and implementing educational interventions and activities for overcoming the organizational barriers since physicians are reported to be less cooperative and facilities are inadequate for EBP implementation in the primary healthcare centers. Finally, future studies should be conducted using a Saudi Arabic version of the BARRIERS scale to obtain highly accurate perceptions of the primary healthcare nurses.

### Limitations of the Study

The utilization of a convenience sample is one of the limitations, which restricted the generalization of results to other populations outside the present study. Hence, the results may not be representative of all nurses in the primary healthcare centers in Saudi Arabia. Moreover, respondents who were willing to participate but had a poor understanding of the English language might be a barrier to implementing EBP, which was not investigated by the instrument used in this study. This study has another limitation. The assessment of the computer knowledge and skills of the respondents about the proper way to search the literature for a specific topic related to nursing practice were not taken into consideration. 

## Figures and Tables

**Table 1:** Nurses’ Perceived Barriers to Implementing EBP (*n* = 284).

BARRIERS Scale Items	Mean	SD
**Adopter Subscale**	**2.87**	**0.65**
8th rank. The nurse feels the benefits of changing practice will be minimal.	3.10	1.05
9th rank. The nurse is unaware of the research.	3.09	0.98
14th rank. There is not a documented need to change practice.	3.02	1.24
16th rank. The nurse does not feel capable of evaluating the quality of the research.	3.01	1.16
20th rank. The nurse sees little benefit for self.	2.93	1.02
26th rank. Nurse is isolated from knowledgeable colleagues with whom to discuss the research.	2.81	1.16
28th rank. The nurse does not see the value of research for practice.	2.69	1.29
29th rank. The nurse is unwilling to change/try new ideas.	2.31	1.15
**Organization Subscale**	**3.14**	**0.66**
1st rank. The nurse feels results are not generalizable to own setting.	3.39	1.05
2nd rank. The facilities are inadequate for implementation.	3.25	0.94
3rd rank. Physicians will not cooperate with implementation.	3.24	0.99
5th rank. Nurse does not feel she/he has enough authority to change patient care procedures.	3.17	1.01
10th rank. The nurse does not have time to read research.	3.09	1.04
11th rank. There is insufficient time on the job to implement new ideas.	3.07	1.25
17th rank. The administration will not allow implementation.	2.99	1.24
21st rank. Other staff are not supportive of implementation.	2.92	1.15
**Innovation Subscale**	**2.98**	**0.72**
6th rank. Research reports/articles are not published fast enough.	3.12	1.05
7th rank. The literature reports conflicting results.	3.11	1.07
12th rank. The conclusions drawn from the research are not justified.	3.05	1.15
15th rank. The research has not been replicated.	3.02	1.05
22nd rank. The research has methodological inadequacies.	2.89	1.09
27th rank. The nurse is uncertain whether to believe the results of the research.	2.72	1.18
**Communication Subscale**	**2.96**	**0.66**
4th rank. The relevant literature is not compiled in one place.	3.23	1.06
13th rank. The research is not reported clearly and readably.	3.03	1.05
18th rank. The research is not relevant to the nurse’s practice.	2.96	1.10
19th rank. Research reports/articles are not readily available.	2.95	1.11
23rd rank. Implications for practice are not made clear.	2.87	1.09
24th rank. Statistical analyses are not understandable.	2.84	1.15
25th rank. The amount of research information is overwhelming.	2.84	1.09

**Table 2:** Differences of BARRIERS Scale Raw Scores and Nurses’ Demographic Characteristics (*n* = 284).

Groups		*n*	Mean	SD	*t*	*p*-Value
**Age**						
	34 years old and below	156	83.78	17.45	1.36	0.19
	Above 34 years old	128	89.30	14.67		
**Gender**						
	Male	75	89.81	13.00	2.13	0.15
	Female	209	85.00	17.02		
**Level of Education**						
	Diploma in Nursing	189	89.20	14.92	0.75	0.39
	Bachelor’s Degree in Nursing	95	81.14	17.08		
**Job Position**						
	Staff Nurse	191	86.64	15.57	2.04	0.16
	Nurse Manager and Assistant Nurse Manager	93	84.52	18.94		
**Years of Experience**						
	12 years or less	161	84.24	16.52	1.21	0.23
	More than 12 years	123	88.92	14.26		
**Evidence-based Training (EBP) Training**						
	Yes	120	83.30	14.43	1.28	0.26
	No	164	88.44	17.08		

**Table 3:** Results of Regression Analysis on the BARRIERS Subscales (*n* = 284).

Predictor Variables	Unstandardized Coefficients		Standardized Coefficients	*t*	*p* Value	(95% Confidence Interval)	
	B	Standard Error	Beta			Upper	Lower
**Adopter Subscale**							
Age	−0.114	0.120	−0.123	−0.950	0.34	0.123	−0.351
Gender	−0.220	0.093	−0.210	−2.357	0.02 *	−0.035	−0.405
Level of Education	−0.285	0.095	−0.297	−3.012	0.003 **	−0.097	−0.472
Job Position	−0.141	0.108	−0.117	−1.309	0.19	0.073	−0.355
Years of Experience	0.062	0.125	0.067	0.498	0.62	0.309	−0.185
Evidence-based Training (EBP) Training	−0.009	0.083	−0.009	−0.105	0.92	0.155	−0.173
**Organization Subscale**							
Age	−0.053	0.109	−0.068	−0.493	0.62	0.162	−0.269
Gender	−0.081	0.085	−0.092	−0.957	0.34	0.087	−0.249
Level of Education	−0.037	0.086	−0.046	−0.436	0.66	0.132	−0.207
Job Position	−0.051	0.098	−0.050	−0.519	0.60	0.143	−0.244
Years of Experience	0.037	0.113	0.048	0.331	0.74	0.261	−0.187
Evidence-based Training (EBP) Training	−0.076	0.075	−0.096	−1.012	0.31	0.073	−0.224
**Innovation Subscale**							
Age	0.196	0.125	0.211	1.567	0.12	0.443	−0.052
Gender	−0.052	0.097	−0.050	−0.539	0.59	0.140	−0.245
Level of Education	−0.165	0.098	−0.172	−1.674	0.10	0.030	−0.360
Job Position	−0.005	0.112	−0.004	−0.041	0.97	0.218	−0.227
Years of Experience	−0.142	0.130	−0.153	−1.094	0.28	0.115	−0.400
Evidence-based Training (EBP) Training	0.122	0.086	0.131	1.416	0.16	0.293	−0.049
**Communication Subscale**							
Age	−0.055	0.119	−0.062	−0.463	0.64	0.181	−0.291
Gender	−0.073	0.093	−0.072	−0.787	0.43	0.111	−0.257
Level of Education	−0.152	0.094	−0.165	−1.618	0.11	0.034	−0.339
Job Position	−0.273	0.107	−0.235	−2.548	0.01 **	−0.061	−0.486
Years of Experience	−0.035	0.124	−0.039	−0.282	0.78	0.211	−0.281
Evidence-based Training (EBP) Training	−0.021	0.082	−0.024	−0.260	0.80	0.142	−0.184

* Significance level at *p* < 0.05. ** Significance level at *p* < 0.01.

## Data Availability

The data presented in this study are available on request from the corresponding author.
